# *TCF7L2 *rs7903146 variant does not associate with smallness for gestational age in the French population

**DOI:** 10.1186/1471-2350-8-37

**Published:** 2007-06-25

**Authors:** Stéphane Cauchi, David Meyre, Hélène Choquet, Samia Deghmoun, Emmanuelle Durand, Stefan Gaget, Cécile Lecoeur, Philippe Froguel, Claire Levy-Marchal

**Affiliations:** 1 CNRS, 8090-Institute of biology, Pasteur Institute, Lille, 59000 France; 2 INSERM, U690, Robert Debré Hospital, Paris, 75019 France; 3 Genomic Medicine, Hammersmith Hospital, Imperial College London, UK

## Abstract

**Background:**

In adults, the *TCF7L2 *rs7903146 T allele, commonly associated with type 2 diabetes (T2D), has been also associated with a lower body mass index (BMI) in T2D individuals and with a smaller waist circumference in subjects with impaired glucose tolerance.

**Methods:**

The present association study aimed at analyzing the contribution of the rs7903146 SNP to smallness for gestational age (SGA) and metabolic profiles in subjects with SGA or appropriate for gestational age birth weight (AGA). Two groups of French Caucasian subjects were selected on birth data: SGA (birth weight < 10^th ^percentile; n = 764), and AGA (25^th ^< birth weight < 75^th ^percentile; n = 627). Family-based association tests were also performed in 3,012 subjects from 628 SGA and AGA pedigrees.

**Results:**

The rs7903146 genotypic distributions between AGA (30.7%) and SGA (29.0%) were not statistically different (allelic OR = 0.92 [0.78–1.09], p = 0.34). Family association-based studies did not show a distortion of T allele transmission in SGA subjects (p = 0.52). No significant effect of the T allele was detected on any of the metabolic parameters in the SGA group. However, in the AGA group, trends towards a lower insulin secretion (p = 0.03) and a higher fasting glycaemia (p = 0.002) were detected in carriers of the T allele.

**Conclusion:**

The *TCF7L2 *rs7903146 variant neither increases the risk for SGA nor modulates birth weight and young adulthood glucose homeostasis in French Caucasian subjects born with SGA.

## Background

Evidence has accumulated that smallness for gestational age (SGA) children have long-term adult health consequences including obesity, type 2 diabetes (T2D), hypertension, coronary artery disease and stroke [1]. This increased risk of later adult disease is likely to be, at least in part, a consequence of an early and persistent insulin resistance although other mechanisms affecting beta-cell function are not excluded [2,3]. In non-pathological conditions, the fetal growth results from complex interactions of maternal and fetal genes with environmental factors such as maternal nutrition and smoking and placental function. Evidence for a genetic contribution for SGA has been reported [4,5] but few genes, associated with diabetes have been reported to also influence birth weight (BW) such as the *INS *VNTR locus [6-8] and the *GCK *gene [9-12]. The *TCF7L2 *rs7903146 polymorphism has been consistently associated with T2D and is probably the causative ancestral allele [13]. In adults, this SNP has been also associated with a lower body mass index (BMI) in T2D individuals [14] as well as with a smaller waist circumference in subjects with impaired glucose tolerance [15].

As O'Rahilly *et al*. recently suggested that the study of *TCF7L2 *should require the analysis of cohorts ascertained for insulin resistance [16], we investigated the relative contribution of the rs7903146 T allele to SGA by comparing 764 individuals with an appropriate for gestational age (AGA) BW (25^th ^< BW < 75th percentile) and 627 SGA subjects (BW < 10^th ^percentile.). Because the mother's genotype may also influence fetal growth, mother-child pairs (n = 361 for SGA and n = 215 for AGA) were also studied. Family-based association tests were then performed in 3,012 subjects from 628 SGA and AGA pedigrees. Birth weight was also analyzed in 845 obese children (BMI = 97^th ^percentile), prone to macrosomia at birth and genotyped for the rs7903146 variant. Finally, we tested the effects of the T allele on birth and adult metabolic parameters in SGA and AGA individuals.

## Methods

### Haguenau case/control cohort

French Caucasian subjects born between 1971 and 1985 were identified from a population-based registry encompassing more than 20,000 births in the metropolitan area of the city of Haguenau, France. Only singletons were included. Gestational age was determined from the date of the mother's last menstrual period and by physical examination during pregnancy, confirmed by ultrasound measurements when available (> 80%). Case-control association tests were performed on two unrelated groups, selected on birth data derived from the local reference curves drawn for gender and gestational age: SGA (birth weight < 10th percentile; n = 627 subjects; 294 men and 333 women) and AGA (birth weight between 25th and 75th percentile; n = 764 subjects; 369 men and 395 women).

Familial association tests were performed on 3,012 individuals from 628 pedigrees, among which 744 children with SGA, and 1,128 children with AGA. All individuals from the case-control study were included in the familial study.

Birth parameters, consisting of birth weight, length, and ponderal index, were collected from the registry. At a mean age of 22 yr, all subjects underwent a medical examination to assess anthropometric parameters (weight, height, waist to hip ratio). We assessed the pancreatic beta-cell function by HOMA beta-cell (HOMA-B) index, as the product of the fasting plasma insulin level (μU/ml) and 20, divided by the fasting plasma glucose level (mmol/l) minus 3.5. To estimate insulin resistance, HOMA-IR was calculated as the product of the fasting plasma insulin level (μU/ml) and the fasting plasma glucose level (mmol/l), divided by 22.5. Fasting insulin, HOMA-B and HOMA-IR were then converted into natural logarithm (Ln) value to normalize the distribution. Total cholesterol, high-density lipoprotein (HDL)-cholesterol, and triglyceride concentrations were measured fasting.

The study protocol was reviewed and approved by the faculty ethics committee, and all subjects and parents gave signed written consent.

### Obese children cohort

All individuals were French Caucasians and were recruited using a multimedia campaign run by the "Centre National de la Recherche Scientifique" (CNRS), UMR8090. All subjects were obese children (n = 845, sex ratio (m/w) = 407/438, age = 11 ± 3 years, BMI z-score = 6.1 ± 2.9, birth weight = 3,448 ± 549 g). The study protocol was approved by the local ethic committee and an informed consent was obtained from each subject before participating in the study.

### Genotyping methods

High-throughput genotyping of the s7903146 variant was performed using the TaqMan^® ^SNP Genotyping Assays (Applied Biosystems, Foster City, Calif. USA). The PCR primers and TaqMan probes were designed by Primer Express and optimized according to the manufacturer's protocol. There was a 98% genotyping success rate and the genotyping error rate was assessed by sequencing 384 control and 384 T2D individuals and by re-genotyping a random 10% sample. No difference was found with the first genotyping results, thus the genotyping error rate was 0%.

### Statistical methods

Tests for deviation from Hardy-Weinberg equilibrium (HWE) and for association were performed with the De Finetti program [17]. Considering that the model of inheritance of the rs7903146 T allele has been found to be additive in the French population [14], we only analyzed allelic odds ratios. Given a 30% allelic frequency for the rs7903146 T allele and a frequency of 10% of SGA, the power of the case-control and family studies was then > 75% to observe an odds ratio = 1.2. Family-based association tests were performed by TDT methods implemented in the FBAT software. For the mother-child pairs, the effect of the T allele on birth weight was tested by linear regression model adjusted for the child's gender and gestational age. For that analysis, the power was > 80% to observe a birth weight variation of 80 g and 60 g in the AGA and SGA groups, respectively. A Student's T-test was applied to compare quantitative traits between SGA and AGA, without taking into account the rs7903146 genotype. Quantitative traits were then measured by linear regression models. These models took known covariables into account: gender and gestational age when testing for birth weight, birth length and ponderal index; age at examination and gender when testing for adult weight, adult height, body mass index (BMI), and waist to hip ratio; age at examination, gender, BMI, when testing for total plasma cholesterol, HDL, triglyceride, fasting glucose, fasting insulin, HOMA-B, HOMA-IR. The power was 80% to observe a variation in fasting glucose concentration of 0.01 g/l and to observe a variation in Ln(HOMA-B) of 0.09 units in the SGA and AGA groups. All tests were two-tailed, and p < 0.05 was considered to be statistically significant. No Bonferroni corrections were applied due to insufficient power. All p values were two-tailed. SPSS 14.1 software (SPSS Inc., Chicago, IL, USA) was used for general statistical analyses.

## Results

### Association studies

**Figure 1:**
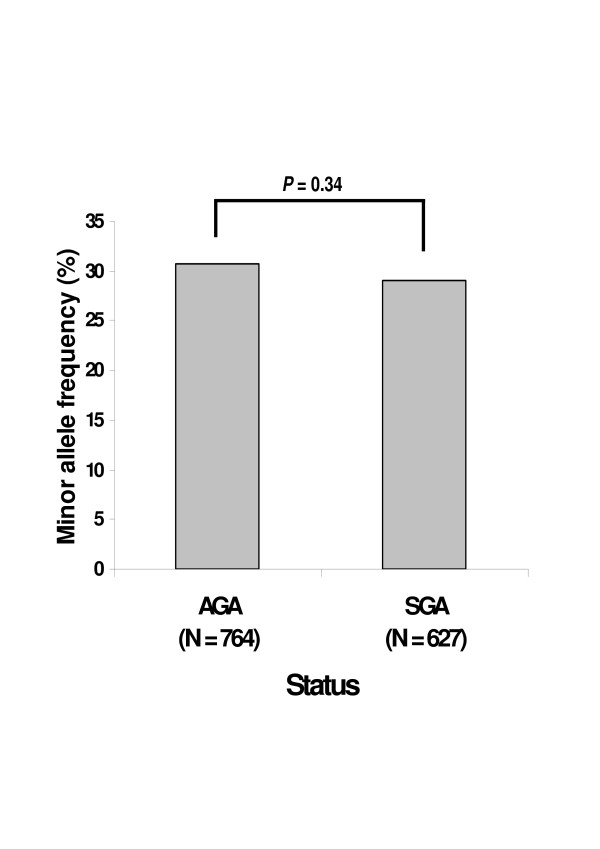
***TCF7L2 *rs7903146 T allele does not associate with SGA**. The T allele frequencies were not significantly different between the SGA and AGA groups (Allelic OR = 0.92 [0.78–1.09], *P *= 0.34). SGA: Smallness for Gestational Age. AGA: Appropriate for Gestational Age birth weight.

Comparison of the rs7903146 T allele frequencies in AGA (30.7%; 368 C/C, 323 C/T and 73 T/T) and SGA (29.0%; 319 C/C, 252 C/T and 56 T/T) are presented in Figure 1. The genotypic distributions between the two groups were in Hardy-Weinberg equilibrium (p = 0.86 for AGA and p= 0.56 for SGA) but were not statistically different (allelic OR = 0.92 [0.78–1.09], p = 0.34). Then the respective effects of mother's and child's genotypes on birth weight were tested by linear regression models (Table 2). No differences in birth weight were found within the 361 mother-child pairs of the SGA group (p = 0.93) and within the 215 mother-child pairs of the AGA group (p= 0.59). Family association-based studies did not show a distortion of T allele transmission in SGA subjects (48.5% for the T allele *vs *51.5% for the C allele, p = 0.52). In 845 obese children (BMI = 97^th ^percentile), we tested the contribution to BW of the rs7903146 genotype by linear regression and after adjustments for gestational age and gender. In this sample, 404 children were C/C (BW = 3,466 ± 506 g), 356 were C/T (BW = 3,425 ± 567 g) and 85 were T/T (BW = 3,464 ± 668 g). The genotypic distribution was in Hardy-Weinberg equilibrium and no difference was found between the 3 genotypic groups (p = 0.61)

**Table 2:** Birth weight parameters by mother and child rs7903146 genotypes

**Mother-child pairs**	**AGA by genotype**			**LR**	**SGA by genotype**			**LR**
	**C/C**	**C/T**	**T/T**	**p value**	**C/C**	**C/T**	**T/T**	**p value**
**Mother**	3371 ± 270	3366 ± 229	3447 ± 273	0.21^a^	2623 ± 325	2634 ± 294	2664 ± 238	0.76^a^
**n**	105	94	16		178	155	28	
**Child**	3340 ± 255	3408 ± 246	3409 ± 264	0.59^b^	2644 ± 315	2600 ± 306	2695 ± 217	0.93^b^
**n**	108	87	20		193	140	28	

n: Number of individuals

p value: the birth

LR: Linear regression model adjusted for child's gender and gestational age

^a^: Effect of the maternal genotype

^b^: Effect of the child genotype adjusted for the maternal genotype

### Quantitative trait analysis

We first analyzed birth and adult (at around 22 years of age) metabolic parameters in AGA and SGA, without taking into account the rs7903146 genotype (Table 1). Insulin resistance (assessed by HOMA-IR index) and lipids profile were significantly different between SGA and AGA individuals. Then, we analyzed the effect of the rs7903146 T allele on quantitative traits by birth status and by genotype (Table 1). No significant effect was detected on any of the metabolic parameters in the SGA group. In the AGA group, however, trends towards a lower Ln(HOMA-B) and towards a higher fasting glucose were detected within the carriers of the T allele. No interaction between the birth status (AGA/SGA) and the rs7903146 variant was found in any of the quantitative traits. The absence of interaction between the genotype and the birth status for fasting glucose and Ln(HOMA-B) may be due to a lack of power.

**Table 1:** Effects of the *TCF7L2 *rs7903146 T allele on birth and adult parameters in SGA and AGA individuals

**Quantitative traits**	**AGA**	**SGA**	**T-test**	**AGA by genotype**			**LR**	**SGA by genotype**			**LR**	**Interaction**
	**n = 764**	**n = 627**	**p *value***	**C/C**	**C/T**	**T/T**	**p *value***	**C/C**	**C/T**	**T/T**	**p *value***	**p *value***
^a^**Birth weight (g)**	3366 ± 267	2614 ± 303	**8 × 10**^-306^	3364 ± 268	3376 ± 260	3334 ± 294	0.78	2623 ± 306	2601 ± 309	2622 ± 258	0.64	0.77
^a^**Birth length (cm)**	50.35 ± 1.2	47.68 ± 2.1	**2 × 10**^-152^	50.3 ± 1.21	50.4 ± 1.1	50.3 ± 1.2	0.61	47.8 ± 2.2	47.6 ± 2.0	47.7 ± 2.0	0.80	0.68
^a^**Ponderal index (kg/m**^3^**)**	26.36 ± 1.6	24.19 ± 2.3	**5 × 10**^-79^	26.4 ± 1.7	26.3 ± 1.6	26.17 ± 1.6	0.41	24.2 ± 2.3	24.2 ± 2.4	24.1 ± 2.5	0.84	0.80

**BMI (kg/m**^2^**)**	22.72 ± 3.97	22.51 ± 4.33	0.36	22.7 ± 3.7	227 ± 4.1	22.7 ± 4.5	0.90	22.9 ± 4.5	22.0 ± 3.9	22.6 ± 5	0.66	0.81
**Waist to hip ratio (no unit)**	0.8 ± 0.08	0.81 ± 0.08	0.20	0.8 ± 0.1	0.81 ± 0.1	0.79 ± 0.1	0.43	0.8 ± 0.1	0.8 ± 0.1	0.8 ± 0.1	0.83	0.86
**HDL cholesterol (mmol/l)**	1.43 ± 0.35	1.4 ± 0.36	0.06	1.4 ± 0.3	1.4 ± 0.3	1.5 ± 0.4	0.32	1.4 ± 0.4	1.4 ± 0.3	1.4 ± 0.3	0.68	0.76
**Triglyceridemia (mmol/l)**	1.03 ± 0.53	1.1 ± 0.59	**0.01**	1.0 ± 0.51	1.09 ± 0.55	0.88 ± 0.49	0.33	1.14 ± 0.64	1.04 ± 0.5	1.2 ± 0.61	0.34	0.21
**Fasting glucose (g/l)**	0.86 ± 0.06	0.87 ± 0.07	0.14	0.86 ± 0.07	0.86 ± 0.07	0.88 ± 0.07	**0.002**	0.87 ± 0.07	0.86 ± 0.06	0.88 ± 0.11	0.64	0.13
**Ln [Fasting insulin (mU/l)]**	1.46 ± 0.5	1.53 ± 0.58	**0.03**	1.5 ± 0.5	1.5 ± 0.5	1.4 ± 0.6	0.42	1.6 ± 0.6	1.5 ± 0.5	1.5 ± 0.7	0.48	0.90
**Ln [HOMA-B (no unit)]**	4.24 ± 0.56	4.29 ± 0.6	0.12	4.27 ± 0.55	4.23 ± 0.56	4.13 ± 0.61	**0.03**	4.32 ± 0.64	4.26 ± 0.53	4.23 ± 0.67	0.71	0.27
**Ln [HOMA-IR (no unit)]**	-0.09 ± 0.51	-0.02 ± 0.61	**0.02**	-0.08 ± 0.52	-0.10 ± 0.49	-0.10 ± 0.58	0.73	0.02 ± 0.66	-0.05 ± 0.52	-0.07 ± 0.71	0.52	0.74

SGA: Small Gestational Age

AGA: Appropriate for Gestational Age birth weight

Insul. Index: Insulogenic index

^a^: Birth parameters

T-test: Student's T-test

LR: Linear regression models (every adjustments are presented in *Subjects and Methods*)

Birth status (AGA/SGA) × genotype interactions were presented on the right side of the table

## Discussion

We found no association between the rs7903146 T allele and SGA, either by case-control or family-based association studies. This may be not surprising as the main quantitative phenotypic effect previously associated with the T allele, at risk for T2D, was only a modest decrease of insulin secretion [14,15,18]. Fetal circulating insulin levels positively correlate with birth weight [19], and therefore, any impaired fetal insulin secretion may only lead to a small decrease in birth weight and not to the more severe SGA condition. Maternal fasting glucose concentration during pregnancy, is also an important determinant of offspring birth weight by stimulating the release of fetal insulin [20]. However, we found no effect of mother's genotype on offspring's birth weight, neither by itself nor by interaction with child's genotype. The lack of association of *TCF7L2 *variation with birth weight was also supported by the analysis of another cohort of French children ascertained by further development of early onset obesity. Interestingly, trends towards a lower insulin secretion and a higher fasting glycaemia were detected in young AGA adults carrying the rs7903146 T allele, in accordance with previous data [14,15,18]. However, the observation of young SGA adults revealed no significant effect of T allele on glucose homeostasis, suggesting that insulin resistance may influence some consequences of this genetic variant, as recently suggested by Watanabe *et al*. [21]. Further studies remain necessary in other ethnic groups and in general populations in order to definitely rule out any effect of *TCF7L2 *on birth weight.

## Conclusion

In conclusion, we showed that the *TCF7L2 *rs7903146 variant neither increases the risk for SGA nor modulates birth weight and young adulthood glucose homeostasis in French Caucasian subjects born with SGA.

## Abbreviations

T2D: Type 2 diabetes

BMI: Body mass index

SGA: Smallness for gestational age

AGA: Appropriate for gestational age birth weight

OR: Odds ratio

HOMA: Homeostasis model assessment

BW: Birth weight

## Competing interests

The author(s) declare that they have no competing interests.

## Authors' contributions

SC carried out the genetic analyses and drafted the manuscript. DM gave strategic advices and participated in the writing. HC carried out the genotyping experiments. SD participated in the design of the study. ED participated in the design of the database. SG participated in the design of the database. CL gave statistical advices. PF coordinated the study. CLM conceived the study, and participated in its design and coordination. All authors read and approved the final manuscript.

## Pre-publication history

The pre-publication history for this paper can be accessed here:


